# Structures and Bioactive Properties of Myrtucommulones and Related Acylphloroglucinols from Myrtaceae

**DOI:** 10.3390/molecules23123370

**Published:** 2018-12-19

**Authors:** Rosario Nicoletti, Maria Michela Salvatore, Pasquale Ferranti, Anna Andolfi

**Affiliations:** 1 Council for Agricultural Research and Economics, Research Centre for Olive, Citrus and Tree Fruit, 81100 Caserta, Italy; rosario.nicoletti@crea.gov.it; 2 Department of Agriculture, University of Naples ‘Federico II’, 80055 Portici, Italy; ferranti@unina.it; 3 Department of Chemical Sciences, University of Naples ‘Federico II’, 80126 Naples, Italy; mariamichela.salvatore@unina.it

**Keywords:** myrtucommulone, acylphloroglucinols, Myrtaceae, plant extracts, biological activities

## Abstract

Myrtaceae are a group of plants that include a number of renowned species used in ethnomedicine in many areas worldwide. Their valuable therapeutic properties have stimulated a fruitful research activity addressed to the identification of the bioactive components of their extracts yielding a great diversity of terpenes; polyphenols; and other exclusive products. Among the latter, starting with the discovery of myrtucommulone A from myrtle (*Myrtus communis*), a series of structurally-related acylphloroglucinol compounds have been characterized from several species that represent the basic active principles to be considered in view of possible drug development. Aspects concerning chemical and biological properties of these products are reviewed in the present paper.

## 1. Introduction

Myrtle (*Myrtus communis*) is a typical shrub of maquis and coastal bushes native of the Mediterranean area and Western Asia. It is well-known in traditional medicine, and for centuries its leaves and berries have found ethnomedical application in the treatment of several disorders of the digestive apparatus, as well as pulmonary and skin diseases [1,2]. More recently, experimental studies have provided an indication for a broader spectrum of pharmacological and therapeutic effects based on antioxidant, antiviral, antibiotic, antitumor, antidiabetic, hepatoprotective, and neuroprotective properties of extracts from this plant [2,3,4]. Many assorted compounds are considered as the possible bioactive components within the myrtle metabolome, such as terpenes occurring in the essential oils, α-tocopherol, anthocyanes, flavanols, and a series of acylphloroglucinols related to the myrtucommulones [4,5,6,7,8,9,10]. Particularly, the number of the latter compounds is continuously increasing as a result of a recent fruitful research activity carried out on other plant species belonging to the Myrtaceae in several independent laboratories worldwide. Structural aspects and the valuable bioactive properties that are pointed out for these compounds are reviewed in the present paper.

## 2. Biological Sources

The family Myrtaceae includes approximately 6000 species in 132 genera, with a wide distribution in tropical and warm-temperate regions of the world [11]. Until recently, these species were grouped in the two subfamilies of the Myrtoideae, including species with fleshy fruits and the Leptospermoideae, whose members produce dry capsules; however, this traditional arrangement has been disrupted in the current taxonomic schemes based on phylogenetic analysis resulting from DNA sequencing [12].

Historically relevant and well-known for its already-introduced multiple usage, *M. communis* represents the type species, and probably the best studied with reference to its biochemical properties. However, investigations in the field are developing more and more, enlarging the panorama of secondary metabolites produced by the Myrtaceae [2,8,13,14,15]. Particularly considered are aspects concerning the antibiotic properties, which have even led to proposing the use of some species in soil sanitization [16]. In this respect, a leading position pertains to the myrtucommulones, originally extracted from myrtle leaves [5,17]. More refined analytical studies have later shown their presence in fruits [7], which preludes the possible dietary intake of these products following the use of berries in gastronomy and in the preparation a digestive liquor typical of Sardinia [18,19].

After the pioneering reports concerning *M. communis*, compounds belonging to this class have been reported in species from other genera of Myrtaceae spread in the Australasian supercontinent. More particularly, species of *Callistemon*, which are now incorporated in the Linnaean genus *Melaleuca* [20], *Angophora, Baeckea*, *Corymbia*, *Eucalyptus*, *Kunzea*, *Lophomyrtus*, *Rhodomyrtus*, and *Syncarpia*, while *Myrciaria dubia* is endemic to the neotropical region (Table 1).

## 3. Structures and Chemical Properties

Within the large family of phloroglucinols [21], a series of alike natural products have been reported from plant species belonging to the Myrtaceae which are characterized by a molecular structure that is built on a phloroglucinol nucleus coupled with one or more syncarpic acid residues (Table 1). Myrtucommulone A (**1**), 4,4′-[(2,4,6-trihydroxy-5-isobutyryl-1,3-phenylene)bis(2-methylpropylidene)]bis(5-hydroxy-2,2,6,6-tetramethylcyclohex-4-ene-1,3-dione), represents the founder product of this series [17].

Despite the use of an inconsistent nomenclature based on the plant species from which the different compounds were originally extracted, all of these structures are clearly related to the myrtucommulone skeleton, and they most likely are assembled through common biosynthetic pathways. A plausible scheme considers the residues of isobutyrylphloroglucinol and isobutylidenesyncarpic acid deriving from the intramolecular Claisen reaction of a polyketidic intermediate obtained from three residues of malonyl CoA and one of isobutyryl CoA [45,53]. In particular, the cyclic intermediate 2-isobutyrylcyclohexane-1,3,5-trione is thought to form isobutyrylphloroglucinol through a double geminal dimethylation, passing through the formation of flavesone [45]. In this hypothesis, the syncarpic acid residue itself is derived from acylphloroglucinol. On the other hand, it has been previously reported that tetramethylation of the polyketidic chain is followed by intramolecular cyclization to form isobutylidenesyncarpic acid [54]. The latter standpoint is supported by what has been assessed for the biosynthesis of several well-known compounds, such as 5-methylorsellic acid [53].

Acylphloroglucinol and substituted syncarpic acid (one or two residues) components are probably coupled via the Michael reaction to form dimeric and trimeric structures [45]. This reaction might be non-enzymatic because the carbon between these residues may lead to the formation of a couple of enantiomers (without further chiral centers) or epimers (with further chiral centers). In fact, in a few cases the enantiomeric composition of myrtucommulones and related compounds is not fully defined. More thorough assessments in this respect are also desirable in view of ascertaining whether or not the bioactivity of myrtucommulones depends on chirality of the molecules.

By means of derivatization, CD spectroscopy and enantiomeric analysis, it has been shown that natural myrtucommulone A is a mixture of three stereoisomers, a racemate, and a meso form in a 1:1 ratio c.a., while myrtucommulone B (**2**) and nor-semimyrtucommulone (**34**) are both racemates. Furthermore, many isomeric forms may occur due to a tautomeric equilibrium of the enolized syncarpic moiety [42].

Further intramolecular reactions could involve the phenolic or ketonic group to form, respectively, pyranic or furanic rings. Prenylation has also been reported for compounds from this class [25,27,45,49,52].

On account of such a wide extent of structural variation, a convenient discussion on the properties of oligomeric acylphloroglucinols should be based on their grouping in subclasses, depending on the number of structural units and the eventual occurrence of additional cycles, such as furan and pyrane. In this respect we distinguished eight homogeneous groups whose members further differ upon the variation in the acyl functionalities occurring in the different moieties (e.g., isobutyryl, isovaleryl, methylbutyryl, and hexanoyl substituents).

**Figure 1:**
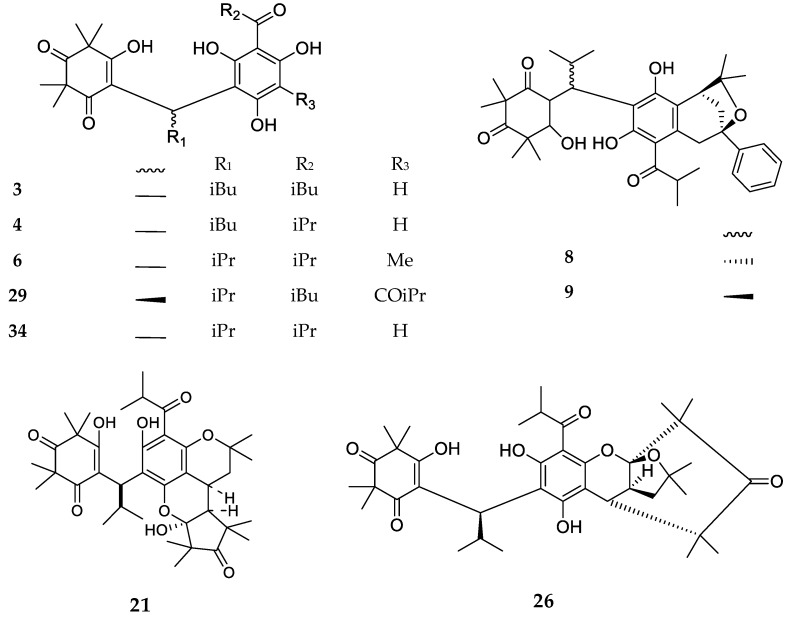
Structures of compounds of the dimeric type.

Compounds in the first subclass (Figure 1) present a dimeric structure, modeled on semimyrtucommulone (**6**) and two closely related compounds (**3**–**4**) reported from *Kunzea* species. Other compounds in this group present additional cyclic structures. In particular, the bullataketals (**8**–**9**) have a phenyl-oxabycyclooctane system, myrtucommulone J (**21**) is characterized by a dipyrancyclopentanone moiety, while myrtucommuacetalone (**26**) contains an unprecedented bridged furochromene moiety.

**Figure 2:**
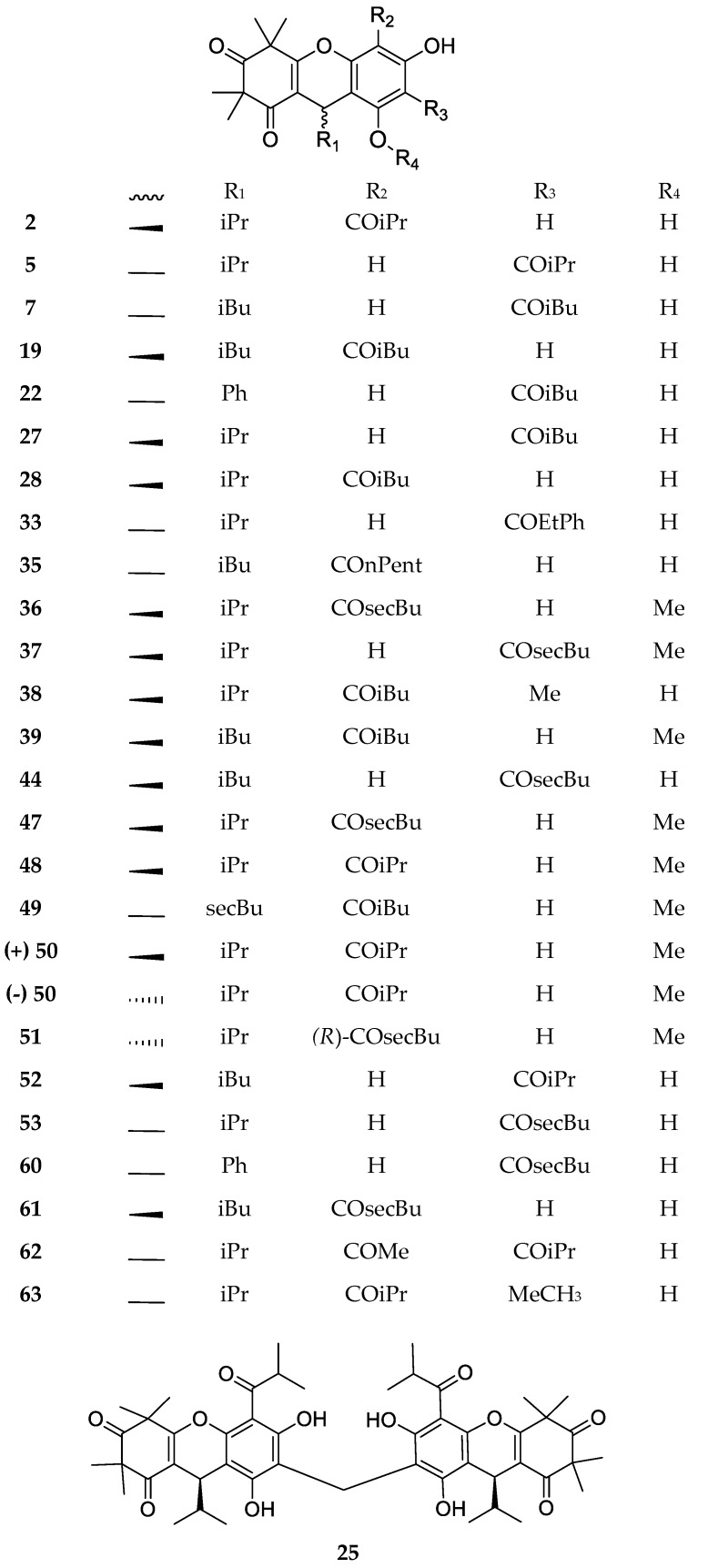
Structures of compounds of the dimeric-monopyrane type.

The dimeric-monopyrane skeleton is shared by over one-third of the compounds examined in this review, which mainly differ in the assortment of the acyl functionalities (Figure 2). This group includes compounds that exhibit a methylated phenolic group on the phloroglucinol residue. Interestingly, myrtucommulone M (**25**) consists in two myrtucommulone B (**2**) moieties that are linked together through a methylene bridge to form a symmetrical structure.

**Figure 3:**
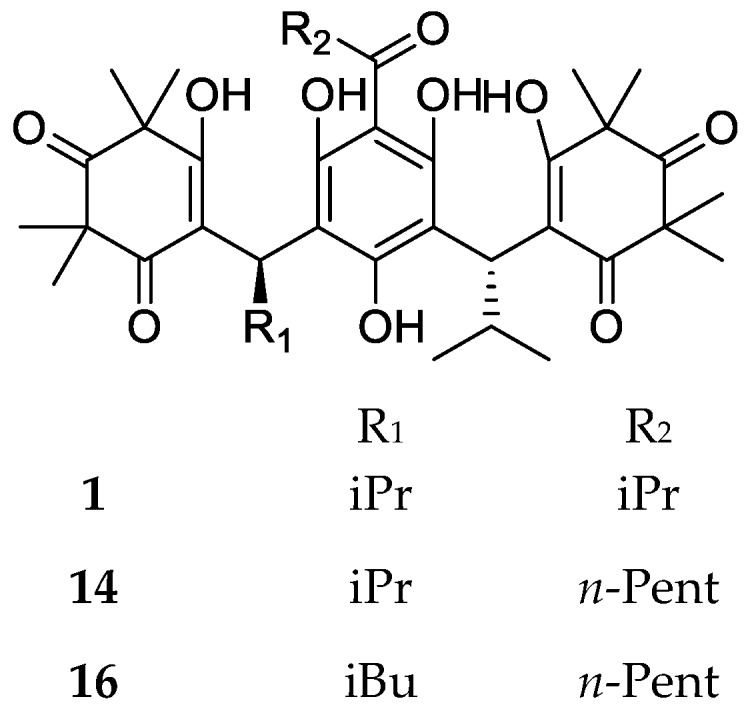
Structures of trimeric compounds. Compound **1** is reported as (*R*,*R*)-stereoisomer.

The founder compound, myrtucommulone A (**1**), and the related myrtucommulones F (**14**) and H (**16**), presenting a hexanoyl residue on the phloroglucinol ring, are characterized by a trimeric structure (Figure 3). This kind of skeleton can be modified by additional cyclization, with the formation of mono and dipyranic analogues that are separated in the following subclasses.

**Figure 4:**
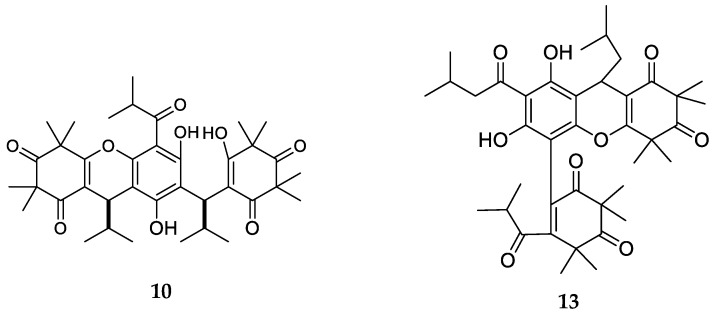
Structures of compounds of the trimeric-monopyrane-type.

Myrtucommulone C (**10**), with a trimeric-monopyrane structure (Figure 4), was isolated as single stereoisomer presenting an isobutanoylic residue. Eucalyptone G (**13**) is also a member of this subclass.

**Figure 5:**
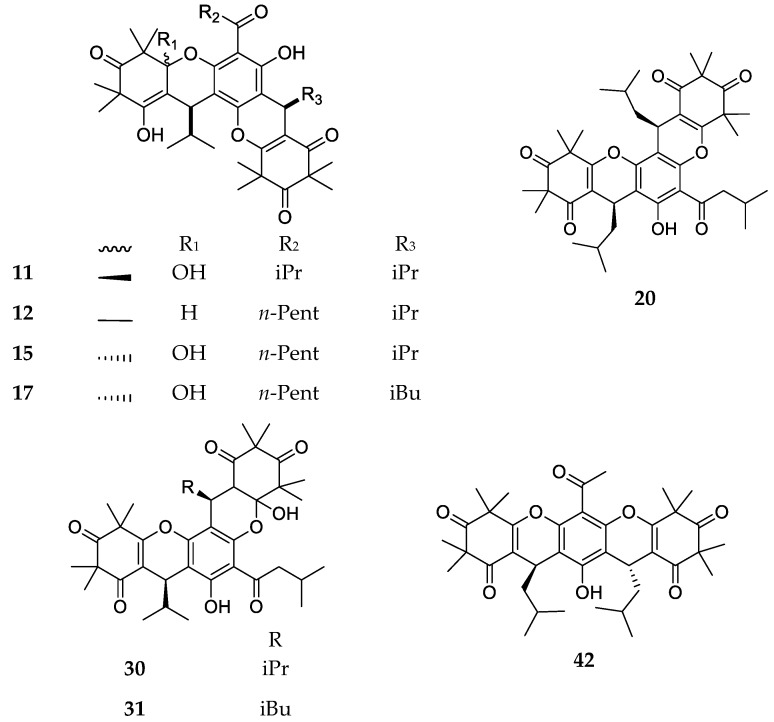
Structures of compounds of the trimeric-dipyrane type.

In products of the trimeric-dipyrane-type (Figure 5), the presence of a pentacyclic structure can be observed where cycles may have an orientation that is similar to pentacene in the case of tomentosone C (**42**), while it is similar to pentaphene for other compounds (**11**, **12**, **15**, **17**, **20**, **30**, **31**). The different orientation is due to diverse phenolic and enolic/ketonic groups that are involved in the formation of pyranic rings of the trimer intermediate.

**Figure 6:**
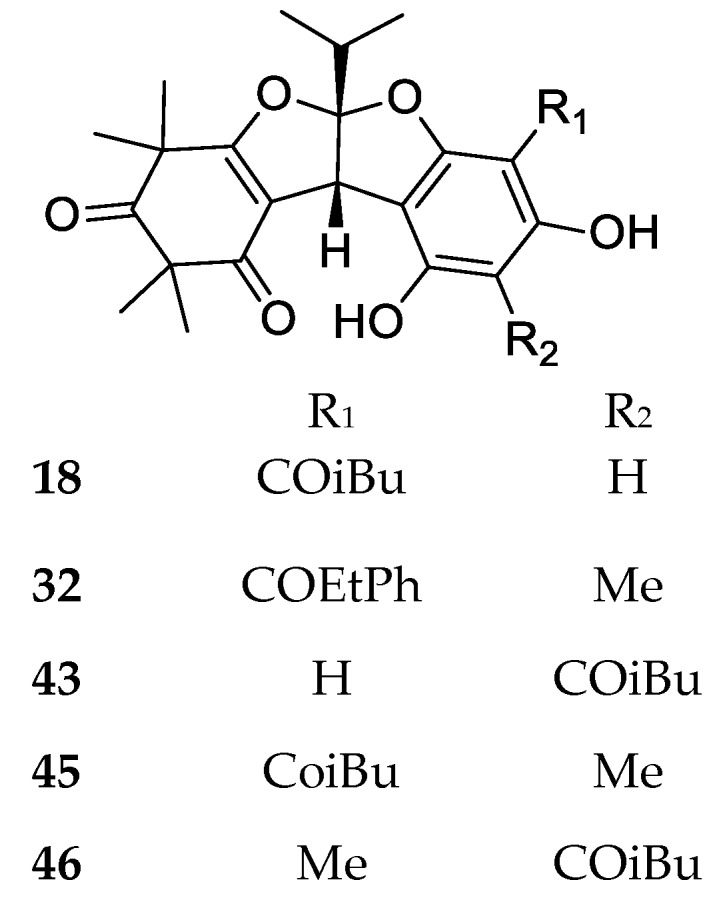
Structures of compounds of the dimeric-bisfurane type.

Rhodomyrtosone A (**18**) from *R. tomentosa* is the first natural product possessing a bisfurane fused ring (Figure 6). The dimeric compound **3** may represent its possible biosynthetic precursor, based on oxidation of the isobutyl side chain followed by the formation of benzofuran via cyclization and dehydration. Afterwards, several related compounds (**32**, **43**, **45**, **46**) have been characterized from other species in the Myrtaceae, indicating a possible wider occurrence of this peculiar structure.

**Figure 7:**
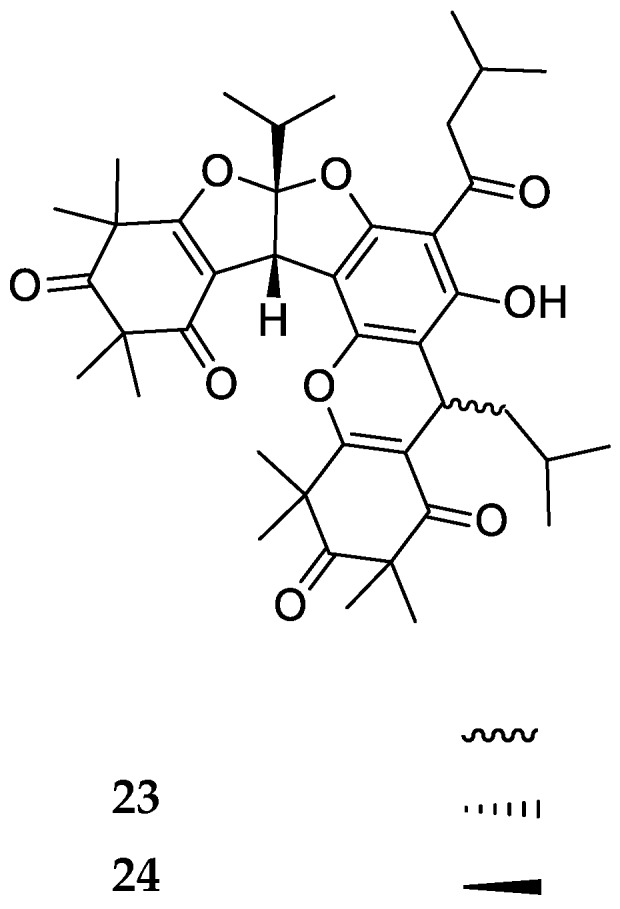
Structures of compounds of the trimeric-bisfurane-pyrane-type.

Tomentosones A and B (**23**, **24**) are two epimers possessing a novel hexacyclic ring system (Figure 7) whose structures present a bisfuranic group and a hexacyclic ring.

**Figure 8:**
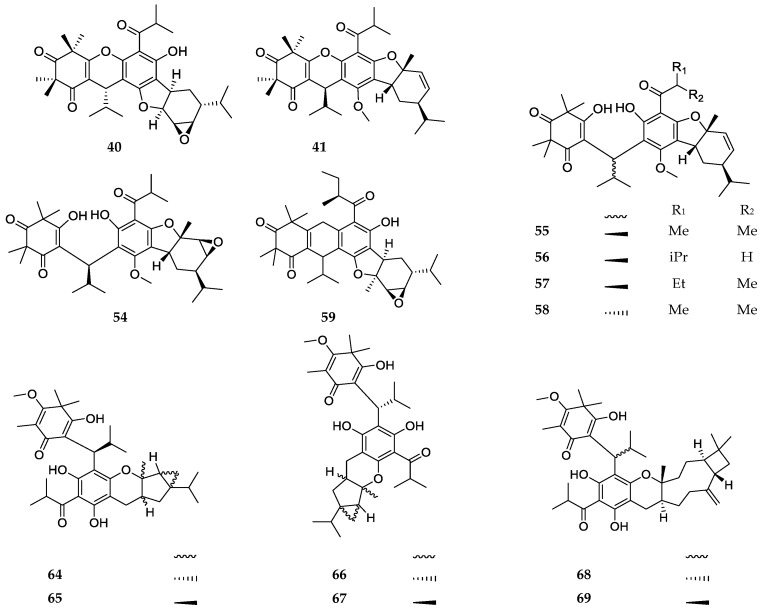
Structures of compounds of the terpene-adduct type.

Callistrilones (**40**, **41**, and **54**–**59**) represent the first syncarpic-phloroglucinol-monoterpene compounds that were isolated from a natural source. These compounds are characterized by the presence of a residue similar to phellandrene, which is fused through a furan ring to the phloroglucinol unit (Figure 8). Other compounds belonging to this class are the baefrutones, four of which (**64**–**67**) show the presence of an iridane skeleton, while **68** and **69** are sesquiterpene adducts.

The available literature concerning Myrtaceae also reports the existence of products that are not classifiable as acylphloroglucinol oligomers, and are hence not included in this review. Particularly, monomers of either acylphloroglucinol (e.g., callisalignene A–C [25], xanchryone A–D [55], operculatol A–B [56]) or syncarpic acid (e.g., myrtucommulone K [35,57], callistiviminene A–O [58]), and flavonoids conjugated to a syncarpic acid residue (e.g., kunzeanones A–C [59], myrtocummunines A–D [51]).

A huge laboratory activity has been carried out on the synthesis of phloroglucinol compounds [60]. As an answer to the rising interest for pharmaceutical applications of myrtucommulones and related compounds, in the last decade several independent approaches have been developed in order to synthesize compounds belonging to this class. In particular, myrtucommulone A was first obtained from commercially available precursors [61], and later through stereoselective synthesis [62,63]. Other analogs of the series have been synthetically obtained (Table 2), particularly in the last couple of years, which possibly preludes further achievements in this respect in the short term.

## 4. Other Biological Sources

**Figure 9:**
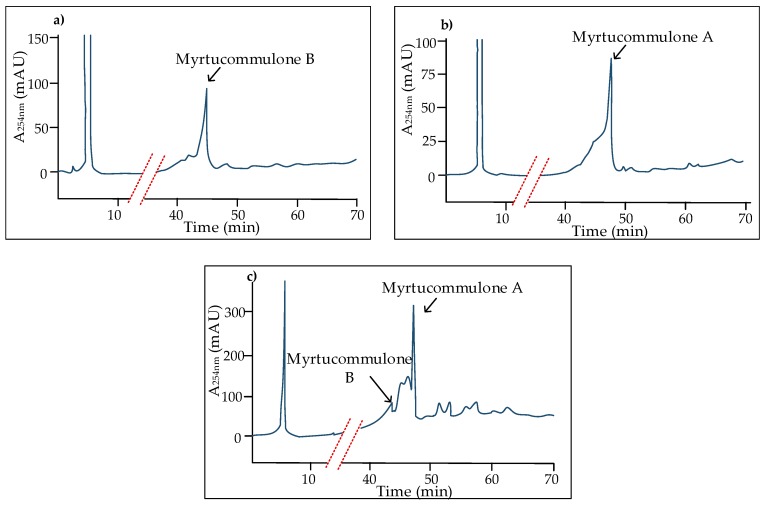
Detection of myrtucommulones A and B through HPLC-DAD analysis in culture extracts of endophytic fungi isolated from *M. communis*. (**a**) A1304B (*N. australe*); (**b**) A1306A (*H. asterinum*); and, (**c**) M15M2B (*N. italica*). Methods for culturing, extraction and chromatography have been previously reported [73].

Following recent discoveries concerning a number of valuable plant-derived drugs that have been also detected as fermentation products of endophytic fungal strains [72], myrtucommulones A and D (**1**, **11**) were extracted from the culture filtrates of a strain of *Neofusicoccum australe* endophytically associated with myrtle [73]. This finding represents the start point for new search terms addressed to a comparative elucidation of the genetic base of myrtucommulone biosynthesis, and possible applicative opportunities for more economic production to be exploited in view of drug development. Actually, while our laboratory investigations are in progress, we can anticipate that this extraordinary aptitude is shared with more endophytic strains isolated from myrtle in several locations (Figure 9). A few of these strains have been taxonomically identified and were found to belong to infrequent species, such as *Neosetophoma italica, Neocucurbitaria cava, Colletotrichum karsti,* and *Helminthosporium asterinum*. Following this preliminary assessment concerning myrtle, more evidences in this respect may be expected if metabolomic investigations are extended to endophytic microorganisms from other species in the Myrtaceae.

Finally, quite interesting is the finding of myrtucommulone I (**17**), together with syncarpic acid and some more identified and unidentified alkylated phloroglucinols in propolis of the Australian stingless bee *Tetragonula carbonaria*, which has been taken into account to explain the antibacterial properties of this bee product [74].

## 5. Biological Activities

### 5.1. Antibacterial Activity

Myrtucommulones A and B (1–2), the founder compounds in this review, were preliminarily characterized for their antibiotic activity in agar plate diffusion assays against Gram-positive bacteria, namely *Staphylococcus aureus, Staphylococcus epidermidis*, *Bacillus subtilis, Bacillus pumilus, Enterococcus faecalis, Corynebacterium diphtheriae,* and *Corynebacterium xerosis* [75]. Similar assays provided concurrent indications in this respect for a few more products, such as the bullataketals (8–9) [31], myrtucommulones C–E (10–12) [33], and eucalyptone G (13) [29]. Afterwards, effectiveness against Gram-positive bacteria has been repeatedly reported for other related compounds, and circumstantiated with details concerning their minimum inhibitory (MICs) (Table 3). Conversely, assays against Gram-negative bacteria have been generally unfruitful, with a few questionable exceptions reporting inhibitory effects against *Escherichia coli* for rhodomyrtone A (7) [28,46], eucalyptone G [29], isomyrtucommulone B (5) [25], and callistenone H (38) [48]. Effects against Gram-negative species other than *E. coli*, such as *Pseudomonas aeruginosa, Salmonella typhi,* and *Shigella flexneri*, were reported for myrtucommulones C–E (10–12) [33].

Particularly interesting is the activity against multi-resistant bacterial strains, especially methicillin-resistant *S. aureus* (MR*Sa*) and vancomycin-resistant *E. faecalis*, exhibited by products, such as callistrilone A (**40**) [45], and rhodomyrtone A (**7**), which did not induce resistance even after 45 passages in vitro [87]. Moreover, the latter product has recently displayed notable effects against both cell division and spore formation in *Clostridium difficile* [83].

Mechanisms of antibacterial activity have been quite thoroughly investigated in the case of **7**. Gene assays and proteomic profiling experiments in *B. subtilis* indicate that the cytoplasmic membrane is the main target of this compound. In *S. aureus,* it was reported to decrease the membrane potential at low doses, and to cause the release of ATP and cytoplasmic proteins. Local membrane damage was confirmed through lipid staining, and the protective effect displayed by saturated fatty acids was explained in terms of counteractive mending. It can be speculated that resistance to **7** by Gram-negative bacteria is due to the reduced penetration of the product through the outer membrane, and its neutralization by lipopolysaccharides. Moreover, interferences in the proteome and metabolome of *Streptococcus pneumoniae* were documented after exposure to **7**, consisting in a reduction of the levels of two enzymes (glycosyltransferase and glucose-1-phosphate uridylyltransferase) and of the uridine diphosphate derivatives of glucose, glucuronic acid, and *N*-acetyl-d-galactosamine, which participate in the synthesis of the capsule; as a matter of fact, a reduction in capsule size was confirmed through a colorimetric assay and electron microscopy [89].

Exposure of MR*Sa* to subinhibitory concentrations of **7** revealed a significant modulation of gene expression. Prominent changes involved genes encoding essential proteins for metabolic pathways and processes, such as membrane function, ATP-binding cassette transportation, and metabolism of amino acids, lipoproteins and nucleotides. Although the amino acid content of peptidoglycan in rhodomyrtone-treated MR*Sa* did not differ significantly from the control, data gathered on genes involved in the biosynthesis of amino acids and the diaminopimelate pathway indicate that peptidoglycan represents a target for bioactivity of this compound [91]. Moreover, proteome analyses in MR*Sa* revealed that exposure to subinhibitory concentrations of **7** affects the expression of several major functional classes of whole cell proteins, which act as surface antigens and virulence factors, or are involved in cell wall biosynthesis, cell division, oxidative stress, and various metabolic pathways. Transmission electron micrographs confirmed that **7** causes morphological and ultrastructural alterations in the bacterial cells, affecting the cell wall with abnormal septum formation and ensuing cell lysis [79].

The protein secretome was also investigated in a representative clinical MR*Sa* isolate, where the immunodominant antigen A, the staphylococcal secretory antigen, and other antigenic proteins involved in cell wall hydrolysis were downregulated after treatment with a subinhibitory concentration of **7**. Ribosomal and cytoplasmic proteins, such as glycerol phosphate lipoteichoic acid synthase and the stage V sporulation protein G (SpoVG), were found in the treated sample, while glycerophosphoryl diester phosphodiesterase, and another lipase precursor were absent. Finally, the finding of several cytoplasmic proteins in the supernatant of the treated cultures indicated impairment in the cell wall synthesis [80].

Again, the proteomic approach was followed in assays carried out with *Streptococcus pyogenes.* Various enzymes associated with important metabolic pathways, including alcohol dehydrogenase, glyceraldehyde-3-phosphate dehydrogenase, Xaa-His dipeptidase, ornithine carbamoyltransferase, putative *O*-acetylserine lyase, enolase (2-phosphoglycerate dehydratase), fructose-bisphosphate aldolase, and cysteine synthase, were strongly affected in a clinical isolate treated with **7** at half the MIC. Moreover, a series of known virulence factors, such as glyceraldehyde-3-phosphate dehydrogenase, CAMP factor, and exotoxin C, were downregulated [84]. It was also found that **7** reduces the synthesis of staphyloxanthin, a pigment promoting resistance to reactive oxygen species (ROS) whose shortage increases the bacterial susceptibility to H_2_O_2_ and singlet oxygen killing [92]. Furthermore, it could reduce biofilm formation by *S. aureus* and *S. epidermidis*, ensuing a reduction in the transcription signals of biofilm-related genes, with a different autolysin profile detectable in the treated cells [77,78]. Besides formation, effects on the disorganization of established biofilms have been recently documented in assays carried out on *Propionibacterium acnes* [86].

Another documented molecular effect of **7** consists in the competitive binding of the tubulin homologue protein FtsZ. In fact, conformational changes in this main bacterial cell division protein were observed in both the (S)- and (R)- binding states of **7**. The compound reduced FtsZ polymerization by 36% and inhibited guanosine triphosphatase activity by up to 45%. However, at inhibitory concentrations, the compound had no effect on FtsZ localization in *B. subtilis*, and cells did not elongate after treatment. Higher concentrations of **7** affected the localization of FtsZ and of its membrane anchor proteins FtsA and SepF, showing that it did not specifically inhibit FtsZ but rather impaired multiple divisome proteins. Cell morphology was sometimes modified to a bean-like shape, possibly implying that the compound may also target cell wall synthesis, or maintenance [93].

At a more applicative level of investigation, it has been observed that subinhibitory concentrations of **7** affect the pathogenicity of oral bacteria (*S. aureus, Streptococcus mutans*) by impairing their adherence to both buccal epithelial cells and a polystyrene support in in vitro assays [84]. Moreover, **7** in a liposomal encapsulated preparation has been proposed for the treatment of bovine mastitis based on observations that were carried out in a bovine udder epidermal tissue model demonstrating remarkable effects against adhesion and invasion of the bacterial agents (*S. aureus* and *S. epidermidis*) [88].

Within a general framework of consistent antibacterial properties, data gathered in Table 3 emphasize that activity may appreciably change among the structural analogues. As an example, the higher efficacy of callistenone A (**27**) in comparison with its B isomer (**28**) is reported to depend on the point of attachment of the isovaleryl side chain [39]. This is confirmed by specific studies based on synthetic analogues, providing indication that the acyl tail of myrtucommulones and related compounds is a prerequisite for the antibacterial properties, and that its affinity for lipids is critical for activity more than its spatial dimension [94,95]. Additional clues in this respect also derive from assays concerning cytotoxicity, which in the case of tomentodiones is potentiated by an isobutyryl chain [50].

### 5.2. Bioactivities against Other Microorganisms and Viruses

The bioactivity of myrtucommulones and related acylphloroglucinols has been also investigated on fungi, with general negative results deriving from assays on species such as *Candida albicans, Cryptococcus neoformans, Microsporum gypseum,* and *Saccharomyces cerevisiae* [27,39].

Conversely, there are positive indications concerning antimalarial properties. In fact, antiplasmodial activity in the nanomolar range was reported for myrtucommulone A (**1**), and at a lower extent for semimyrtucommulone (**6**) [5,96]. Watsonianone B (**32**) displayed a potent activity against strain 3D7 of *Plasmodium falciparum* (IC_50_ 0.289 μM), particularly against the young ring stages, coupled with selectivity towards a human embryonic kidney cell line (HEK 293) [40]. Strong effects against the chloroquine resistant strain Dd2 (IC_50_ 0.10 μM) and low toxicity towards HEK 293 also characterize rhodomyrtosone F (**35**) [42], while a more moderate activity was assessed for rhodomyrtone A (**7**) against both 3D7 and Dd2 (IC_50_ 1.84 µM and 4.00 µM, respectively) [30]. Moreover, tomentosone A (**23**) inhibited the growth of both chloroquine resistant and sensitive strains (IC_50_ 1.49 μM and 1.0 μM, respectively), while its B analogue (**24**) was significantly less active [37]. Structural comparisons indicated that the syncarpic acid moiety is essential for antiplasmodial activity [97]. Additional data concerning antiplasmodial properties of the above products extracted from the flowers of *Angophora woodsiana* are reported in another paper by the same research group [30].

Antiviral effects were displayed in vitro by the mixture of compounds **3**–**4**, so far only extracted from two *Kunzea* species, based on the inhibition of the cytopathic effects of *Herpes simplex* type 1 (HSV-1) and Polio type 1 viruses [26]. Moderate effects in similar assays against HSV-1 have been also reported for callistrilones H–I (**38**–**39**) [49].

### 5.3. Antioxidant and Anti-Inflammatory Activities

Besides the possible applications in the treatment of infective diseases based on the above-reported effects, multiple observations concerning consistent antioxidant and anti-inflammatory properties have resulted from investigations that were carried out in several laboratories, representing an indication of a potential for a therapeutic use in the treatment of a series of disorders, ranging from allergopathies to cardiovascular diseases. With reference to the latter, myrtucommulone A (**1**) and semimyrtucommulone (**6**) were reported to exert powerful antioxidant properties during the degradation of cholesterol, preserving the LDL form from oxidative damage induced by copper ions, and inhibiting the increase of oxidative products deriving from polyunsaturated fatty acids [98,99]. At micromolar concentrations both compounds suppressed eicosanoid biosynthesis in vitro and in vivo by directly inhibiting cyclooxygenase (COX)-1 and 5-lipoxygenase. Moreover, they were successful in preventing the mobilization of Ca^2+^ in polymorphonuclear leukocytes, mediated by G protein signalling pathways, with the first compound acting at lower concentrations, and suppressed the formation of ROS and the release of elastase [100].

Binding affinity to the thyrotropin-releasing hormone (TRH) receptor-2, which is known to play a role in the phosphoinositide metabolism and is regarded as a potential therapeutic target to treat pain, was also reported for myrtucommulones A, D, and F–I (**1**, **11**, **14**–**17**) at micromolar concentrations [23]. After experiments carried out on both a human lung adenocarcinoma cell line (A549) and in a cell-free assay based on microsomal preparations of A549 cells stimulated with interleukin (IL)-1β, myrtucommulone resulted to be the first natural product to inhibit microsomal prostaglandin synthase-1 that efficiently suppresses prostaglandin formation without significant inhibition of cyclooxigenases, hence without displaying the typical side effects of non-steroidal anti-inflammatory drugs [101]. Furthermore, **1** exerted anti-inflammatory effects in the pleurisy model. In particular, a reduction was observed in the exudate volume, leukocyte numbers, lung injury, and neutrophil infiltration, and in a series of more specific effects mediated by enzymes and cytokines [102].

The interest for a potential therapeutic use of rhodomyrtone A (**7**) is also based on consistent properties that may prevent or delay the progression of inflammation in skin diseases, such as psoriasis. After stimulating human skin organ cultures with TNF-α and IL-17A to mimic skin inflammation, **7** significantly decreased inflammatory gene expression and the secretion of inflammatory proteins. Particularly, it inhibited TNF-induced extracellular signal–regulated kinases (ERK), c-Jun *N*-terminal kinases (JNK), the mitogen-activated protein (MAP) kinase p38, and phosphorylation of the NF-κB transcription factor p65, suggesting that it acts by modulating MAP kinase and NF-κB signalling pathways. Moreover, it reversed imiquimod-induced skin hyperplasia and epidermal thickening in mice [103]. The potential of **7** as an anti-psoriasis agent is further increased by its property to inhibit proliferation and to induce growth arrest and apoptosis in HaCaT keratinocytes [104]. The expression of pro-inflammatory molecules, including IL-1β, IL-6, TNF-α, and inducible nitric oxide synthase (iNOS) was enhanced in THP-1 monocytes that were stimulated with a high dose of heat-killed MR*Sa*. In contrast, monocytes stimulated with lower doses did not express these cytokines. However, in monocytes stimulated with heat-killed MR*Sa* at low doses, **7** significantly increased the expression of pro-inflammatory mediators, IL-6 and iNOS, and displayed some anti-inflammatory activity by reducing TNF-α expression. Treatment with **7** also significantly upregulated the expression of key pattern recognition receptor proteins (TLR2 and CD14). The ability of **7** to eliminate the resistant bacteria was observed within 24 h after treatment, following enhancement of the expression in monocytes of MR*Sa* recognition receptors, which possibly improved MR*Sa* clearance by modulating pro- and anti-inflammatory cytokine responses [105].

The possible application of the anti-inflammatory and immunomodulatory properties of **7** has been suggested in aquaculture as a result of observations carried out in vitro on head kidney macrophages of the rainbow trout (*Oncorhynchus mykiss*). In fact, exposure to **7** (1 μg mL^−1^) induced changes in the expression of genes involved in innate immune and inflammatory responses, particularly with reference to pro-inflammatory cytokines (IL-1β, IL-8, TNF-α), anti-inflammatory cytokines (IL-10, TGF-β), the antioxidant enzyme glutathione peroxidase 1, and other inducible enzymes (iNOS, COX-2, arginase). Co-exposure of **7** with lipopolysaccharides led to a downregulation of genes encoding for some of the above inflammation-related products and a reduction in ROS levels [106].

Myrtucommuacetalone (**26**) exhibited a significant inhibitory effect against production of nitric oxide, a ROS generated by NADPH oxidases in human peripheral blood phagocytes whose excess is associated with the pathogenesis of various diseases, such as colitis, diabetes, septic shock, and ischemic neuronal damage. Moreover, it inhibited the proliferation of T-cells, which is a relevant effect for the prevention or treatment of autoimmune disorders, such as Parkinson’s disease, rheumatoid arthritis, and diabetes [38]. Additionally, inhibitory effects on nitric oxide production after lipopolysaccharide stimulation in murine macrophage cells (RAW 264.7) have been documented for baefrutones A–D (**64**–**67**) [52], and for tomentodione T (**61**) and rhodomyrtosones B, G, and I (**19**, **43**, **22**) [50].

### 5.4. Cytotoxic and Antiproliferative Activities

Apart from the diverse implications arising from the above properties, the observation of a pro-apoptotic effect induced on human cancer cell lines more directly introduces a possible relevance of myrtucommulones and associated acylphloroglucinols as antitumor drugs. Tretiakova et al. [107] first showed that, at micromolar concentrations, myrtucommulone A (**1**) induces apoptosis in several cancer cell lines, such as PC-3 (androgen-independent prostate carcinoma), LNCaP (androgen-dependent prostate carcinoma), KFR (rhabdomyosarcoma), HL-60 (acute promyelocytic leukemia), MM6 (acute monocytic leukemia), H9 (cutaneous T-cell lymphoma), DLD-1 (colorectal adenocarcinoma), and Jurkat (acute T-cell leukemia). Cell death occurred through the mitochondrial pathway involving the activation of caspase-3, -8, and -9, cleavage of poly(ADP-ribose)polymerase (PARP), release of nucleosomes into the cytosol, and DNA fragmentation. A lower cytotoxic effect was displayed on non-transformed human peripheral blood mononuclear cells and foreskin fibroblasts. Apoptosis appeared to be mediated by the intrinsic pathway, with the loss of the mitochondrial membrane potential in MM6 cells and the release of cytochrome c from mitochondria. Interestingly, Jurkat cells deficient in caspase-9 were resistant to apoptosis, and no processing of PARP or caspase-8 was evident. Conversely, in cell lines that were deficient in either CD95 signalling or caspase-8, myrtucommulone was still able to induce cell death and PARP cleavage.

A more direct indication that **1** induces apoptosis by triggering the intrinsic pathway and directly disrupting the mitochondrial functions resulted in assays carried out on HL-60 cells. In these cells, the compound caused the loss of the mitochondrial membrane potential and suppressed mitochondrial ATP synthesis, consequently inducing the adenosine monophosphate-activated protein kinase (AMPK), an energy sensor involved in apoptosis of cancer cells. More in detail, **1** acts as a protonophore that primarily dissipates the mitochondrial membrane potential through a direct structural interaction, and suppresses the proton motive force that impairs mitochondrial viability and activates AMPK due to lowered ATP levels [108]. The chaperonin heat-shock protein 60 (HSP60) also represents a molecular target of myrtucommulone A, which binds the protein and modulates its mitochondrial functions. Particularly, in a protein refolding assay the compound was found to prevent HSP60-mediated reactivation of denatured malate-dehydrogenase [109].

Myrtucommulone A (**1**) also induced apoptosis in several chronic myelogenous leukemia cell lines (K-562, MEG-01, KBM-5) in consequence of downregulation of anti-apoptotic proteins, as evidenced by nuclear fragmentation and PARP cleavage. Interestingly, the compound displayed differential toxicity, since peripheral blood mononuclear cells from healthy donors that were used as control were unaffected [110].

In further studies carried out on murine breast cancer cells (4T1), **1** was found to trigger apoptosis at micromolar concentrations through both the intrinsic and extrinsic apoptotic pathways. The compound mediated an increased expression of several apoptotic genes, such as Fas, FasL, Gadd45a, Tnf, Tnfsf12, Trp53, and caspase-4. Moreover, the results of a wound healing experiment showed that it is also able to inhibit cancer cell migration [111]. All these effects were enhanced when treatment was operated in combination with epirubicin or cisplatin, evidencing a synergistic effect that could be exploited for setting more effective therapeutic schemes [112].

Another relevant effect of **1** that may contrast tumor development consists in a reduction of the expression of endoglin, a membrane glycoprotein that has a crucial role in angiogenesis. Treatment with this product reduced the chondrogenic potential in human mesenchymal stem cell (hMSC) lines, possibly as a consequence of the NF-κB p65 activation, while the adipogenic or osteogenic differentiation was not dramatically affected. The exploitation of these properties could be useful in targeted differentiation studies [113].

It is known that hMSCs can be observed in tissues surrounding tumors, where they could play a role in regulating cancer cell behaviour through paracrine signalling. Therefore, the modulation of their secretome is highly significant in view of attempts to control the disease. **1** was effective in modulating cytokine expression in hMSCs with a decrease of TNF-α, IL-6, IL-8, the vascular endothelial growth factor (VEGF), and the basic fibroblast growth factor (FGF-2), and in reducing the proliferation, migration, and clonogenicity of human bladder (HTB-9) and 4T1 cancer cells [114]. Moreover, when considering the prominent role that is played by the epithelial-mesenchymal transition in cancer progression and metastasis, the ability of **1** to interfere in this process again by modulating signalling pathways and inhibiting phosphorylation of multiple proteins represents a notable therapeutic property [115]. Besides reducing the viability and proliferation in HTB-9 cells, in cancer stem cells the compound downregulated the expression of markers associated to pluripotency and multipotency (i.e., NANOG, OCT-4, SOX-2, SSEA-4, TRA-1-60, CD90, CD73, and CD44), and decreased sphere-forming ability [116].

As an integration to data concerning efficacy, perspectives for a possible pharmaceutical use of myrtucommulones are corroborated by the absence of substantial cytotoxicity for non-malignant cells [107], and evidence of molecular stability in human and rat plasma [117].

Additional indications concerning the inhibitory effects on tumor cells have resulted in several laboratories from assays that were carried out with a number of compounds of the myrtucommulone series. Particularly, antiproliferative activity has been reported for bullataketals A and B (**8**–**9**) on murine leukemia cells (P388) [31], for myrtucommulones A and J (**1, 21**) on another prostate cancer cell line (DU145), HEpG2 (human liver carcinoma) and MT-4 (lymphocytic leukemia) [35], on PC3 and DU145 treated with a mixture of **1** and myrtucommulone D (**11**) [73], on HCT116 (human colorectal carcinoma) treated with **11** and isomyrtucommulone B (**5**) [25], on HeLa (human cervix uteri carcinoma) for tomentodiones S and T (**60**–**61**), rhodomyrtone A (**7**), and rhodomyrtosones A, B, G, and I (**18**, **19**, **43**, **22**) [50].

Rhodomyrtone A (**7**) is cytotoxic for several types of eukaryotic cells, and eryptosis induced in human erythrocytes progresses along with cell shrinkage, membrane blebbing, and phosphatidylserine translocation to the cell surface. The distinctive interactions with the cytoplasmic membrane assimilate **7** to amphipathic products, introducing it as a useful tool in studies on membrane physiology [118]. While confirming that it is responsible for membrane invaginations that form intracellular vesicles trapping a broad range of membrane proteins, Saeloh et al. [119] observe that **7** does not behave as a typical membrane-inserting molecule; in fact, it transiently binds to phospholipid head groups and causes a distortion of lipid packing, which explains membrane fluidization and curvature. However, both the transient binding mode and the ability to form protein-trapping vesicles are unique properties, which are possibly indicative of a peculiar mechanism of action.

More detailed investigations provide further insight in the antitumor properties of **7**, which has been recently characterized as an antimetastatic agent for the treatment of skin cancer cells after studies that were carried out on an epidermoid carcinoma cell line (A431). In fact, at subcytotoxic concentrations the compound reduced migration of tumor cells, as well as their adhesive and invasive ability. At the molecular level it was able to inhibit the focal adhesion kinase (FAK) and phosphorylation of protein kinase B (AKT), c-Raf, ERK1/2, and p38 involved in the downregulation of enzyme activities and the expression of matrix metalloproteinase (MMP)-2 and -9. Moreover, the compound increased the expression of TIMP-1 and TIMP-2, which are inhibitors of MMP-9 and MMP-2, respectively, and inhibited the expression and phosphorylation of NF-κB in a dose-dependent manner [120].

### 5.5. Other Pharmacological Perspectives

The α-glucosidase-inhibitory activity displayed by myrtucommulones C–E (**10**–**12**) could introduce therapeutic application of these compounds in another widespread disease, such as diabetes [33]. Moreover, rhodomyrtosone E (**33**) showed weak effects on the translocation to the plasma membrane of the insulin-responsive glucose transporter 4 (GLUT-4) protein, representing another attractive target for anti-diabetic drug development [41]. Finally, the strong inhibitory activity towards soluble epoxyde hydrolases that is exhibited by myrtucommulone B (**2**) and callistenone B (**28**) could be exploited for the treatment of a variety of pathological conditions [24].

Besides data resulting from specific assays carried out with the purified compounds, the widespread use in ethnomedicine of extracts of plant species in the Myrtaceae represents a reliable guide for the possible pharmaceutical applications of these products [121,122,123]. Based on its consistent antibacterial properties, the use of myrtle extracts has been proposed for the treatment of mild bacterial disorders such as vaginosis [124], and an ethanolic myrtle extract (Myrtacine^®^) has been registered for the treatment of acne lesions whose active principles are claimed to be myrtucommulones A and B [125,126]. For the latter dermatological use, rhodomyrtone A has been proposed in an innovative liposome encapsulation in order to overcome problems deriving from the poor water solubility [127]. The same kind of preparation has also been tested for zootechnical use [88].

## 6. Conclusions

Structures and properties of 69 myrtucommulone-related products that were extracted from plant species belonging to the Myrtaceae during the past 45 years have been reviewed in this paper. Considering that about half of these compounds have been discovered in the last two years, and that attention of the scientific community to the exploitation of natural resources of bioactive products is increasing more and more, it is predictable that their number is going to increase quite quickly in the future. Furthermore, in the aim to study more in detail aspects concerning the relationships between structure and bioactivity of these products, synthetic studies have been recently started in a few laboratories from which quite a high number of analogues have been obtained, indicating that these structural models may be used for the synthesis of novel variants with improved effects [94,95,128,129].

Advances in the exploitation of these valuable products require a more refined capacity to detect their occurrence in plants [130,131,132], and to perform a careful extraction and purification of the effective analogues. Alternative opportunities to obtain these products through controlled fermentation also represent a basic investigational line that is likely to attract the attention of leading actors in the field of drug discovery and development in the near future.

## Figures and Tables

**Table 1:** Myrtucommulones and related compounds reported from plant species belonging to the Myrtaceae. Compounds are listed according to the chronological order of discovery.

Code	Compound Name	Formula, Nominal Mass (U)	Source	Ref.
**1**	Myrtucommulone A	C_38_H_52_O_10_, 668	*Myrtus communis* *Melaleuca citrina* ^1^ *Corymbia scabrida*	[17] [22] [23]
**2**	Myrtucommulone B	C_25_H_32_O_5_, 412	*Myrtus communis* *Melaleuca citrina* ^1^ *Melaleuca salicina* ^1^	[17] [24] [25]
**3**	4-Cyclohexene-1,3-dioxo-5-hydroxy-2,2,6,6-tetramethy1-4-{1-[2,6-dihydroxy-4-methoxy-3-(3-methyl-1-oxo-butyl)phenyl]-3-methylbutyl}	C_26_H_36_O_7_, 460	*Kunzea ericoides* *Kunzea sinclairii*	[26]
**4**	4-Cyclohexene-l,3-dioxo-5-hydroxy-2,2,6,6-tetramethyl-4-{l-[2,6-dihydroxy-4-methoxy-3-(2-methyl-1-oxopropyl)pheny1]-3-methylbutyl}	C_25_H_34_O_7_, 446	*Kunzea ericoides* *Kunzea sinclairii*	[26]
**5**	Isomyrtucommulone B	C_24_H_30_O_6_, 414	*Myrtus communis* *Melaleuca salicina* ^1^ *Myrciaria dubia*	[5] [25] [27]
**6**	Semimyrtucommulone	C_25_H_34_O_7_, 446	*Myrtus communis*	[5]
**7**	Rhodomyrtone A	C_26_H_34_O_6_, 442	*Rhodomyrtus tomentosa* *Eucalyptus globulus* *Angophora woodsiana* *Myrciaria dubia*	[28] [29] [30] [27]
**8**	Bullataketal A	C_37_H_48_O_7_, 604	*Lophomyrtus bullata* *Lophomyrtus obcordata*	[31] [32]
**9**	Bullataketal B	C_37_H_48_O_7_, 604		
**10**	Myrtucommulone C	C_38_H_50_O_9_, 650	*Myrtus communis*	[33]
**11**	Myrtucommulone D	C_38_H_50_O_9_, 650	*Myrtus communis* *Corymbia scabrida* *Melaleuca salicina* ^1^	[33] [23] [25]
**12**	Myrtucommulone E	C_38_H_48_O_8_, 632	*Myrtus communis*	[33]
**13**	Eucalyptone G	C_40_H_52_O_9_, 676	*Eucalyptus globulus*	[29]
**14**	Myrtucommulone F	C_40_H_56_O_10_, 696	*Corymbia scabrida*	[23]
**15**	Myrtucommulone G	C_40_H_54_O_9_, 678		
**16**	Myrtucommulone H	C_41_H_58_O_10_, 710		
**17**	Myrtucommulone I	C_41_H_56_O_9_, 692		
**18**	Rhodomyrtosone A	C_26_H_32_O_7_, 456	*Rhodomyrtus tomentosa* *Angophora woodsiana*	[34] [30]
**19**	Rhodomyrtosone B	C_26_H_34_O_6_, 442	*Rhodomyrtus tomentosa*	[34]
**20**	Rhodomyrtosone C	C_41_H_54_O_8_, 674		
**21**	Myrtucommulone J	C_38_H_52_O_8_, 636	*Myrtus communis*	[35]
**22**	Rhodomyrtosone I	C_28_H_30_O_6_, 462	*Rhodomyrtus tomentosa*	[36]
**23**	Tomentosone A	C_41_H_52_O_9_, 688	*Rhodomyrtus tomentosa*	[37]
**24**	Tomentosone B	C_41_H_52_O_9_, 688		
**25**	Myrtucommulone M	C_49_H_60_O_12_, 840	*Myrtus communis*	[38]
**26**	Myrtucommuacetalone	C_38_H_52_O_9_, 652		
**27**	Callistenone A	C_25_H_32_O_6_, 428	*Melaleuca citrina* ^1^	[39]
**28**	Callistenone B	C_25_H_32_O_6_, 428	*Melaleuca citrina* ^1^ *Melaleuca salicina* ^1^	[39] [25]
**29**	Callistenone C	C_29_H_40_O_8_, 516	*Melaleuca citrina* ^1^	[39]
**30**	Callistenone D	C_39_H_52_O_9_, 664		
**31**	Callistenone E	C_40_H_54_O_8_, 662		
**32**	Watsonianone B	C_31_H_34_O_7_, 518	*Corymbia watsoniana*	[40]
33	Rhodomyrtosone E	C_30_H_34_O_6_, 490	*Eucalyptus citriodora*	[41]
**34**	Nor-semimyrtucommulone	C_24_H_32_O_7_, 432	*Myrtus communis*	[42]
35	Rhodomyrtosone F	C_27_H_36_O_6_, 456	*Syncarpia glomulifera*	[43]
**36**	Callistenone F	C_26_H_34_O_6_, 442	*Melaleuca viminalis* ^1^	[44]
**37**	Callistenone G	C_26_H_34_O_6_, 442		
**38**	Callistenone H	C_26_H_34_O_6_, 442	*Melaleuca viminalis* ^1^ *Melaleuca salicina* ^1^	[44] [25]
**39**	Callistenone I	C_27_H_36_O_6_, 456	*Melaleuca viminalis* ^1^	[44]
**40**	Callistrilone A	C_33_H_42_O_7_, 550	*Melaleuca linearis* ^1^	[45]
**41**	Callistrilone B	C_35_H_46_O_6_, 562		
**42**	Tomentosone C	C_38_H_48_O_8_, 632	*Rhodomyrtus tomentosa*	[46]
**43**	Rhodomyrtosone G	C_26_H_32_O_7_, 456	*Rhodomyrtus tomentosa*	[47]
**44**	Rhodomyrtosone H	C_26_H_34_O_6_, 442		
**45**	Callistenone L	C_27_H_34_O_7_, 470	*Melaleuca viminalis* ^1^	[48]
**46**	Callistenone M	C_27_H_34_O_7_, 470		
**47**	Callistenone N	C_26_H_34_O_6_, 442		
**48**	Callistenone O	C_25_H_32_O_6_, 428		
**49**	Callistenone P	C_27_H_36_O_6_, 456		
**50**	Callisalignone B	C_25_H_32_O_6_, 428	*Melaleuca salicina* ^1^	[25]
**51**	Callisalignone C	C_26_H_34_O_6_, 442		
**52**	Myrciarone A	C_25_H_32_O_6_, 428	*Myrciaria dubia*	[27]
**53**	Myrciarone B	C_25_H_32_O_6_, 428		
**54**	Callistrilone F	C_35_H_48_O_8_, 596	*Melaleuca linearis* ^1^	[49]
**55**	Callistrilone G	C_35_H_48_O_7_, 580		
**56**	Callistrilone H	C_35_H_52_O_7,_ 608		
**57**	Callistrilone I	C_36_H_50_O_7_, 594		
**58**	Callistrilone J	C_37_H_52_O_7_, 608		
**59**	Callistrilone K	C_36_H_48_O_6_, 576		
**60**	Tomentodione S	C_28_H_32_O_5_, 448	*Rhodomyrtus tomentosa*	[50]
**61**	Tomentodione T	C_28_H_32_O_5_, 448		
**62**	6-Methylisomyrtucommulone B	C_25_H_32_O_6_, 428	*Myrtus communis*	[51]
**63**	4-Methylmyrtucommulone B	C_25_H_32_O_6_, 428		
**64**	Baefrutone A	C_35_H_48_O_7_, 580	*Baeckea frutescens*	[52]
**65**	Baefrutone B	C_35_H_48_O_7_, 580		
**66**	Baefrutone C	C_35_H_48_O_7_, 580		
**67**	Baefrutone D	C_35_H_48_O_7_, 580		
**68**	Baefrutone E	C_40_H_56_O_7_, 648		
**69**	Baefrutone F	C_40_H_56_O_7_, 648		

^1^ Species names have been updated according to their current taxonomic status [20].

**Table 2:** Myrtucommulone-related compounds obtained synthetically.

Code	Compound Name	Subclass	Ref.
**1**	Myrtucommulone A	Trimeric type	[61,62,63]
**7**	Rhodomyrtone A	Dimeric-monopyrane	[64,65]
**18**	Rhodomyrtosone A	Dimeric-bisfurane type	[66]
**19**	Rhodomyrtosone B	Dimeric-monopyrane type	[64,65,66]
**21**	Myrtucommulone J	Dimeric type	[67]
**26**	Myrtucommuacetalone	Dimeric type	[67,68]
**40**	Callistrilone A	Terpene-adduct type	[68,69,70]
**55, 58**	Callistrilone G, J	Terpene-adduct type	[71]

**Table 3:** Bioactivity of myrtucommulones and related compounds resulting from assays carried out on Gram-positive bacteria.

	*Bc*	*Bs*	*Cd*	*Ef*	*Ml*	*Pa*	*Sa*	MR*Sa*	*Se*	*Sg*	*Sm*	*Spn*	*Spy*	*Ss*	Ref.
**1**							1–2	0.5							[5]
**2**							7.813								[25]
	8–16						16–32	16–32							[51]
**5**	1.56	0.78					1.56		6.25		3.13				[27]
						0.8									[10]
							0.488								[25]
	1–2						1–2	0.5–1							[51]
**6**							32–64	32							[5]
**7**	0.39	0.39		1.56			0.39	0.39–0.78	0.39	0.19	0.19	0.39	0.39	0.39	[76]
							0.5–1		0.25–1						[77,78]
							2	0.5							[79]
							0.5	0.5							[80]
							0.78				0.39				[81]
	0.5														[82]
			0.62–2.5												[83]
													0.78		[84]
						0.12–0.5									[85,86]
							1.83								[46]
				1–32			0.5–1	0.5–1							[87]
							0.5	0.5							[80]
	0.78	0.78			0.78		0.78		0.78		1.56				[27]
							0.5–1		0.5–1						[88]
												0.5–1			[89]
**11**							1.953	0.975							[25]
**19**				2.5			0.62–1.25	0.62–1.25							[90]
**21**							0.38								[35]
**27**							0.5	1							[39]
**28**							8	8							[39]
**29**							8	8							[39]
**38**							20.3								[48]
**40**				32			16	16							[45]
**41**				64											[45]
**42**							3.66								[46]
**52**	1.56	1.56			1.56		1.56		3.13		3.13				[27]
**53**	1.56	1.56					1.56		3.13		1.56				[27]
**62**	8						8	8–16							[51]

MIC_50_ is reported in µg/mL. Bc = Bacillus cereus; Bs = Bacillus subtilis; Cd = Clostridium difficile; Ef = Enterococcus faecalis or E. faecium; Ml = Micrococcus luteus; Pa = Propionibacterium acnes; Sa = Staphylococcus aureus; MRSa = methicillin-resistant S. aureus; Se = Staphylococcus epidermidis; Sg = Streptococcus gordonii; Sm = Streptococcus mutans; Spn = Streptococcus pneumoniae; Spy = Streptococcus pyogenes; Ss = Streptococcus salivarius.
